# Proteomics and Metabolomics for *In Situ* Monitoring of Wound Healing

**DOI:** 10.1155/2014/934848

**Published:** 2014-08-04

**Authors:** Stefan Kalkhof, Yvonne Förster, Johannes Schmidt, Matthias C. Schulz, Sven Baumann, Anne Weißflog, Wenling Gao, Ute Hempel, Uwe Eckelt, Stefan Rammelt, Martin von Bergen

**Affiliations:** ^1^Department of Proteomics, Helmholtz-Centre for Environmental Research-UFZ, Permoserstraße 15, 04318 Leipzig, Germany; ^2^University Center of Orthopedics and Trauma Surgery, University Hospital “Carl Gustav Carus”, TU Dresden, Fetscherstraße 74, 01307 Dresden, Germany; ^3^Department of Oral and Maxillofacial Surgery, University Hospital “Carl Gustav Carus”, TU Dresden, Fetscherstraße 74, 01307 Dresden, Germany; ^4^Department of Metabolomics, Helmholtz-Centre for Environmental Research-UFZ, Permoserstraße 15, 04318 Leipzig, Germany; ^5^Institute of Pharmacy, Faculty of Biosciences, Pharmacy and Psychology, University of Leipzig, 04103 Leipzig, Germany; ^6^Institute of Physiological Chemistry, TU Dresden, Fiedlerstraße 42, 01307 Dresden, Germany; ^7^Department of Biotechnology, Chemistry and Environmental Engineering, Aalborg University, Sohngaardsholmsvej 49, 9000 Aalborg, Denmark

## Abstract

Wound healing of soft tissue and bone defects is a complex process in which cellular differentiation and adaption are regulated by internal and external factors, among them are many different proteins. In contrast to insights into the significance of various single proteins based on model systems, the knowledge about the processes at the actual site of wound healing is still limited. This is caused by a general lack of methods that allow sampling of extracellular factors, metabolites, and proteins *in situ*. Sampling of wound fluids in combination with proteomics and metabolomics is one of the promising approaches to gain comprehensive and time resolved data on effector molecules. Here, we describe an approach to sample metabolites by microdialysis and to extract proteins simultaneously by adsorption. With this approach it is possible (i) to collect, enrich, and purify proteins for a comprehensive proteome analysis; (ii) to detect more than 600 proteins in different defects including more than 100 secreted proteins, of which many proteins have previously been demonstrated to have diagnostic or predictive power for the wound healing state; and (iii) to combine continuous sampling of cytokines and metabolites and discontinuous sampling of larger proteins to gain complementary information of the same defect.

## 1. Introduction

Bone replacement and bone regeneration are currently in the focus of research. Due to the changes in the population pyramid and thus an increase of related comorbidities (e.g., diabetes, vascular diseases) the need for new biofunctional materials increases [1]. The analysis of wound fluid (WF) provides a direct insight into the local extracellular microenvironment of a wound [2]. In the last years, several proteins, especially inflammatory mediators, growth factors and cytokines [3, 4], proteases [5, 6], and oxidative stress related proteins [7, 8], were identified in WF which can be used to characterize and classify the effect on different phases of the wound healing process and thereby can be used as predictive and diagnostic tools. Nevertheless, the underlying mechanisms in wound healing disturbance are not completely understood and so far none of the potential biomarkers for dysregulated wound healing was established in clinical routine diagnostics. Consequently, molecular insights into the factors being involved as well as their interplay are of great interest. Immunoblotting, microbead arrays, and enzyme immunoassays are applied commonly in wound healing studies. However, these techniques require cost-intensive protein-specific antibodies for each analyte of interest and therefore are practically limited to the analysis of a relative small number of selected proteins.

Mass spectrometry based proteomics is one of the promising approaches to gain comprehensive profiling data which can be used for diagnostic purposes or to discover potential molecular diagnostic/prognostic biomarkers which do reflect discrepancies from undisturbed wound healing [9]. Proteomics in combination with, for example, microdialysis has already been used to study fluids from a wide variety of different tissues such as skin [10], brain [11], liver [12], bone [13], and eye [14].

However, the high complexity (several thousand proteins), the low sample amount (few micrograms), the low protein concentration in wound fluids, the presence of salts and metabolites, and especially the high excess of serum proteins compared to other proteins such as mediator proteins are technical challenges which so far limited proteome studies to the coverage of only a few dozen high abundant proteins [15, 16].

Even though several approaches are described, there is still no gold standard method for the reproducible and robust protein extraction from wound fluids.

Microdialysis is one of the established techniques allowing the recovery of a wide range of molecules including low molecular weight proteins which are sampled based on diffusion and ultrafiltration. A semipermeable membrane designed to mimic a blood capillary is inserted into the wound. This probe is continuously perfused with a few microliters per minute flow using a dialysate which has a comparable ionic strength as the wound fluid. Hence, in a minimal-invasive manner a low but representative amount of the complex wound fluid can be collected* in situ* [10, 13, 17, 18].

Alternatively, WF can be sampled discontinuously using wound swapping [19], occlusive dressings [20], porous dextranomer beads [21], capillary tubes, or paper strips [22, 23]. Using these methods the collected amount of protein is higher; however, the wound fluid contains salts as well as metabolites and the protein concentration is quite low and consequently requires additional cleaning and enrichment steps for proteomic analysis [17].

Here we describe an alternative approach using membranes of microdialysis catheters as solid phase extraction material. In this study, we evaluated (i) whether the sampling can be used to collect, enrich, and purify proteins in a sufficient amount for a proteome analysis and whether additional cleaning or enrichment steps are required; (ii) how many and which proteins can be detected; (iii) whether there is a bias regarding the biological or biophysical properties of the identified proteins; and (iv) whether the approach can be applied to perform proteomics and metabolomics of the same samples.

In this study, two different defect scenarios were examined including a soft tissue and a femoral bone defect. The obtained desorbates were highly concentrated, contained up to 100 micrograms of total protein, required no further purification, and were sufficient for a reproducible analysis of more than 500 proteins by in-gel separation and proteolytic digestion followed by liquid chromatography-tandem mass spectrometry (GeLC-MS/MS).

More than 12% (73 proteins) are annotated to be functionally involved in the response to wounding such as metalloproteases, S100 proteins, annexins, complement factors, and several serine proteases and serine protease inhibitors—many of them were already reported to have diagnostic or predictive power for wound healing in diverse defects.

Furthermore it was possible to quantify 163 selected metabolites in the microdialysis dialysates from the same defects.

## 2. Material and Methods

### 2.1. Animal Surgery

Approval for conducting the animal experiments was granted by the local animal care committee (24-9168.11-1/2010-22 and 2010/10). All animals were housed according to the European guidelines for the care and use of laboratory animals (Directive 24.11.1986, 86/609/CEE). Nine male Wistar rats with an average body weight of 300 g were obtained from Janvier (Le Genest Saint Isle, France) and held in the animal care unit for at least 7 days before the experiments.

### 2.2. Femoral Bone Defect

The surgeries were prepared as previously described [13]. Briefly, the rats were anesthetized (ketamine (100 mg/kg body weight, Riemser Arzneimittel AG, Greifswald, Germany) and xylazine (10 mg/kg body weight, Pharma-Partner Vertriebs-GmbH, Hamburg, Germany)) and kept in anesthesia up to 24 h (administration of ketamine/xzlazine through a permanent catheter intraperitoneally every 90–120 min). After shaving and disinfection of the right hind limb, a longitudinal incision of 3 cm was made. The femur was surgically exposed by dissecting the thigh muscles. A five-hole plate (Stryker, Hamburg, Germany) was fixed with four screws (1.5 × 5 mm) on the femur and for one group (femoral bone defect) additionally a 5 mm bone defect using a gigli saw. The microdialysis catheter was either inserted into soft tissue defect (*N* = 4) or the femoral bone defect (*N* = 5). The soft tissues were closed in two layers with absorbable sutures and disinfected again. To avoid hypothermia the rats were covered with thin sheets during the experiment. The rats were killed at the end of the experiment.

The commercially available CMA/20 microdialysis catheter (CMA Microdialysis AB, Solna, Sweden) with a 4 mm polyethylene sulfone membrane and a cutoff of 100 kDa was used for the experiments. The microdialysis probe was primed with an initial flush (5 *μ*L/min) for 15 min. The membrane was perfused with perfusion fluid (PER, 147 mmol/l NaCl, 4 mmol/l KCl, 2.3 mmol/l CaCl_2_, 1% BSA) at a flow rate of 2 *μ*L/min using a CMA402 microdialysis pump (CMA Microdialysis). Samples were collected over 12 h. After catheter explantation and dialysis collection the catheters and dialysates were stored at −20°C.

### 2.3. Targeted Metabolite Quantification of Dialysates

Concentrations of 163 metabolites from cell lysates were determined using a targeted metabolomic approach with the AbsoluteIDQ p150 kit (BIOCRATES Life Sciences AG, Innsbruck, Austria). The 163 metabolites belong to acylcarnitines (41), amino acids (13 proteinogenic + ornithine), hexoses (sum of hexoses—90–95% glucose), and phospho- and sphingolipids such as phosphatidylcholines (77), lysophosphatidylcholines (15), and sphingomyelines (15).

40 *μ*L of the dialysates which were collected over the first 12 h after surgery was diluted 1 : 100 with PBS. The samples were centrifuged at 15,000 g for 2 min and supernatants were prepared afterwards according the manufacturers protocol. Briefly, 10 *μ*L of the cell lysate was pipetted onto a 96-well sandwich filter plate, including stable isotope labeled internal standards. The filters were dried in nitrogen stream, amino acids were derivatized with 5% phenyl isothiocyanate reagent (PITC), and filters were dried again. After extraction of metabolites and internal standards with 5 mM ammonium acetate in methanol, the solution was centrifuged through the filter membrane and diluted with MS running solvent. The final extracts were analyzed by FIA-MS/MS. Methanol and ethanol were purchased in highest purity from Merck (Darmstadt, Germany); ammonium acetate, pyridine, and phenyl isothiocyanate (PITC) were obtained from Sigma-Aldrich (Seelze, Germany).

### 2.4. 1D-SDS-PAGE and Tryptic Digestion

Proteomics analyses of the dialysates as well as of the catheter adsorbates were performed. Microdialysis catheters were boiled in SDS sample buffer (62.5 mM Tris-HCl (pH 6.8), 6% glycerol, 2% SDS, 5% mercaptoethanol, and 0.05% bromophenol blue) for 5 min and subjected together with the resulting protein eluate onto a 1D-SDS-PAGE (4% stacking gel and 5–20% separation gel) according to standard laboratory procedures. The gels were cut into eight slices after staining with Coomassie Brilliant Blue G250 solution.

The dialysates were concentrated and a puffer exchange to 100 mM ammonium bicarbonate buffer was performed by filtration using centrifugal filtration units (molecular weight cutoff 10 kDa, Millipore, Billerica, USA). The samples were dried by vacuum centrifugation and resolubilized using SDS buffer and 30 *μ*g of the proteins extract was separated by 1D-SDS-PAGE and prepared for digestion as described above.

In-gel digestion of all samples was performed as previously described [25]. Briefly, the gel slices were destained with 50% methanol/5% acetic acid. After reduction with 10 mM dithiothreitol, the proteins were alkylated with 100 mM iodoacetamide. In-gel digestion was conducted overnight at 37°C using 50 ng sequencing grade trypsin (Roche Applied Science, Mannheim, Germany) per slice. The resulting peptides were extracted two times from the gel with 5% formic acid and 50% acetonitrile. The combined extracts were evaporated, the residual peptides were dissolved in 20 *μ*L 0.1% formic acid, and the solutions were stored at −20°C until LC-MS/MS analysis.

### 2.5. LC-MS/MS Analysis

A nano-HPLC system (nanoAcquity, Waters, Milford, MA, USA) coupled to a LTQ Orbitrap XL ETD mass spectrometer (Thermo Fisher Scientific, San Jose, CA, USA) via chip based nanoelectrospray ion source (TriVersa NanoMate, Advion, Ithaca, NY, USA) was used for LC-MS/MS analysis as it has previously been described [26]. Peptide elution was conducted using a 120 min gradient (2–40% acetonitrile containing 0.1% formic acid; 300 nl/min). The mass spectrometer automatically switched between full scan MS mode (positive mode,* m/z* 350 to 1,600, *R* = 60,000) and MS/MS acquisition of the six most abundant. Peptide ions exceeding an intensity of 3,000 counts were fragmented within the linear ion trap by collision induced dissociation (isolation width 4 amu, normalized collision energy 35, activation time 30 ms, and activation Q 0.25). A dynamic precursor exclusion of 3 min for tandem MS measurements was applied.

### 2.6. Processing of Data Obtained by LC-MS/MS

LC-MS/MS measurements were analyzed by Proteome Discoverer (Thermo Scientific, version 1.4.0.288). Proteome Discoverer was set up to perform target-decoy database search using the Mascot search engine. A database containing forward and reverse entries of all rat proteins listed in the UniProt database (version 12th Dez 2013) was utilized. Trypsin was set as endoprotease allowing for a maximum of 2 missed cleavages. Carbamidomethylation of cysteine was specified as a fixed modification, whereas oxidation of methionine was specified as variable modification. The protein and peptide identification results were adjusted to a FDR of maximum 1% and a minimum of two peptides considering only first ranked peptides was set for the identification of proteins. Only proteins which were identified in at least two biological replicates were considered. The proteins were quantified by a peak area based label-free quantification using the three most abundant peptides per protein.

### 2.7. Bioinformatic Analysis

GO term enrichment analysis was performed with DAVID Bioinformatics Resources 6.7 [27] (http://david.abcc.ncifcrf.gov). For a cluster analysis all proteins which were detected in the femoral bone and soft tissue defects were mapped to human DAVID IDs using DAVID ID Conversion tool. The proteins which could be mapped were clustered according to the biological processes (GO_BP_FAT) and the cellular compartment (GO_CC_FAT). A subset of the 103 proteins which were annotated as located extracellularly (GO:0005615~extracellular space, GO:0005576~extracellular region, and GO:0044421~extracellular region part) was created. That subset was clustered again according to biological processes (GOTERM_BP_FAT) and uploaded to STRING 9.1 (string-db.org). A protein-protein interaction network was generated based on experimental and database entries. Using the clustering results generated with DAVID the data were clustered according the annotation to the GO terms “homeostasis,” “cell death,” “defense response/inflammation,” “immune response,” and “proteolysis.” For supplementary tables annotations using PANTHER database [28] (http://www.pantherdb.org/) were included. pI values and molecular weights were calculated using the ExPASy tool “Compute pI/Mw” (http://web.expasy.org/compute_pi/).

## 3. Results

### 3.1. Classical* In Situ* Sampling of Metabolites and Proteins by Microdialysis

**Figure 1:**
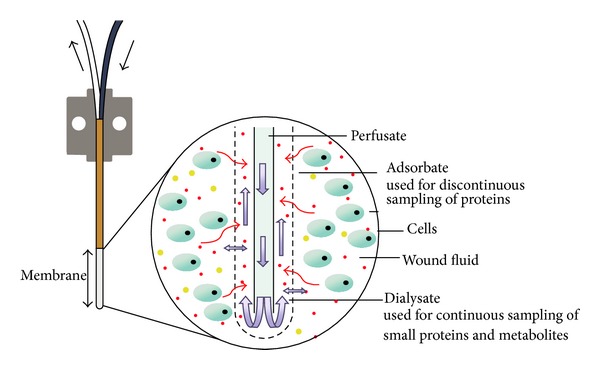
Scheme of the microdialysis setup (modified to CMA Microdialysis AB, Solna, Sweden).

Protein and metabolite sampling using microdialysis catheters (molecular weight cutoff of 100 kDa) was performed with a perfusion flow rate of 2 *μ*L/min (setup is schematically shown in Figure 1). For the quantification of more than 150 selected metabolites including acylcarnitines (41), amino acids (14), hexoses, phosphatidyl- (77), lysophosphatidylcholines, and sphingolipids (15) only 10 *μ*L was required. The median quantification accuracy below 30% could be obtained among the three biological replicates of the femoral bone defect as well as for the soft tissue defect. Detailed results of targeted metabolites demonstrate the potential of a parallel quantification of metabolites (Supplementary Table S1 available online at http://dx.doi.org/10.1155/2014/934848) as well as proteins from the same sample source. The low requirements in terms of sample volume and the quantification in a high-throughput manner offer the potential for a time resolved experiment with sampling periods of every 10 minutes in further investigations.

The quantification of proteins from dialysates was rather inefficient. Only a few but very dominant bands could be observed by 1D-SDS-PAGE and finally 15 proteins could be quantified in at least 3 samples (Supplementary Table 2). Among the quantified proteins high as well as low molecular weight proteins are present (e.g., complement C3 (186 kDa) and ribosomal protein S27a (8.5 kDa)); thus no clear bias against high molecular weight proteins was observed. Nevertheless, even though low amounts of proteins such as complement C3 (activation of complement system) or serpin c1 (regulation of the blood coagulation cascade) could be quantified, which are of high relevance for wound healing, the obtained coverage was highly incomplete.

### 3.2. *In Situ* Sampling of Wound Fluid Proteins by Solid Phase Extraction Using Microdialysis Catheter

While salts and metabolites efficiently pass the dialysis membrane, only minimal amounts of proteins were observed to penetrate the membrane. Moreover proteins also adsorbed to it. As previously was determined, even low molecular weight proteins such as IL-6 (20.8 kDa) or TGF-beta 1 (12.8 kDa) were nearly completely retarded (recovery below 10%) by dialysis membranes with a cutoff of 100 kDa [13]. Anticipating that a significant percentage of the protein amount was adsorbed at the microdialysis catheter material, the catheters' tips were used to extract, enrich, and purify proteins directly from different tissue defects.

Immediately after explanting the adsorbed proteins were eluted and resolved by SDS-PAGE sample buffer, as a valuable side effect, this results in an efficient protein denaturation and reduction of disulfide bonds and thus inhibits proteolysis and prevents degradation. In dependency of the defect up to 100 *μ*g of protein was obtained. The entire sample without further purification was subjected to a 1D-gradient-SDS-PAGE gel.

### 3.3. GeLC-MS Analysis of Extracted Proteins Allowed Coverage of More Than 600 Proteins in Different Defect Scenarios

**Figure 2:**
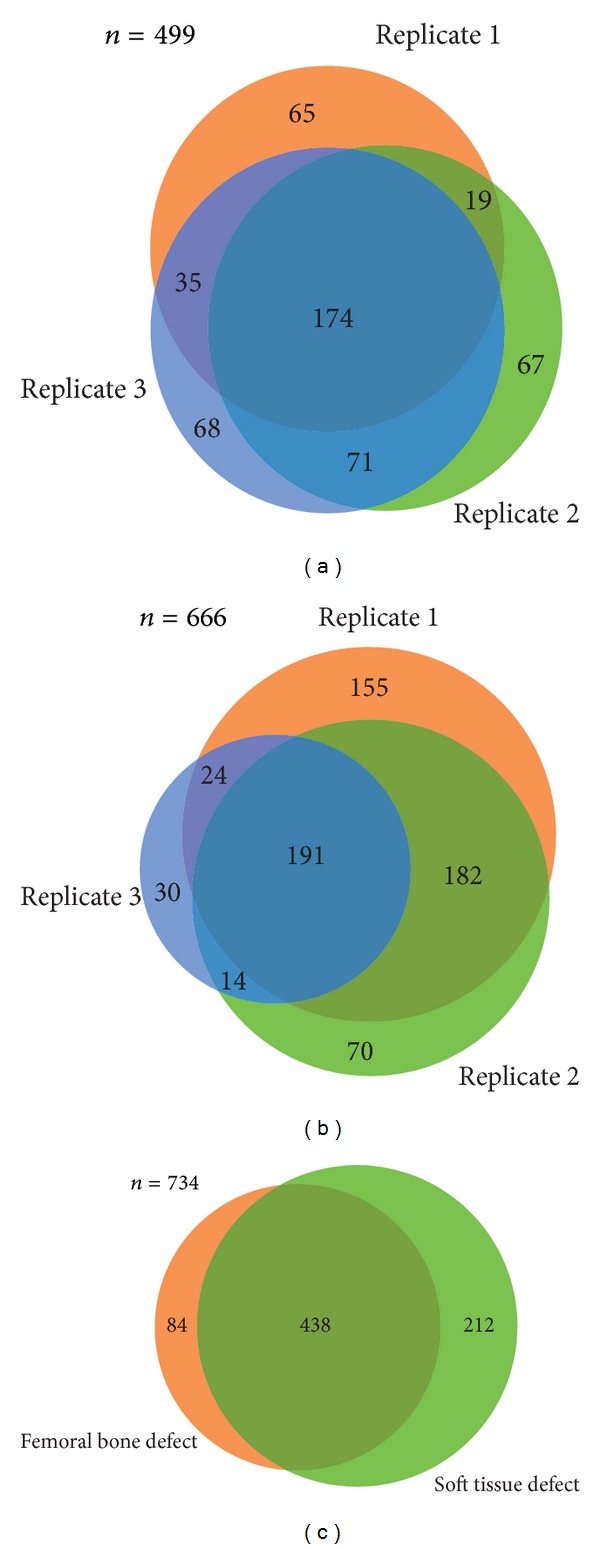
Analysis of the reproducibility of protein identifications between biological replicates and between the different defects. Venn diagram showing the overlap between proteins covered in replicates of the femoral bone defect (a) and soft tissue defect (b), *N* = 3, and (c) between proteins being identified in wound fluids of the three different defects.

Soft tissue and femoral bone defects were studied in 3 and 5 biological replicates, respectively. Gel lanes were cut into 8 slices of approximately equal protein amounts according to the staining intensity to reduce signal suppression by high abundant proteins. Proteins were enzymatically cleaved by in-gel digestion and the derived proteolytic peptide mixtures were analyzed by high performance liquid chromatography-tandem mass spectrometry (LC-MS/MS). Thus, in total 734 proteins could be identified in the WF of the two investigated defects (Supplementary Table 3). 299 were repeatable detected in femoral bone defects whereas 541 were repeatable covered in the soft tissue defect (Figures 2(a) and 2(b)). 438 proteins were identified in the wound fluids of both defect scenarios.

### 3.4. Evaluation of a Bias to pI or MW Ranges

**Figure 3:**
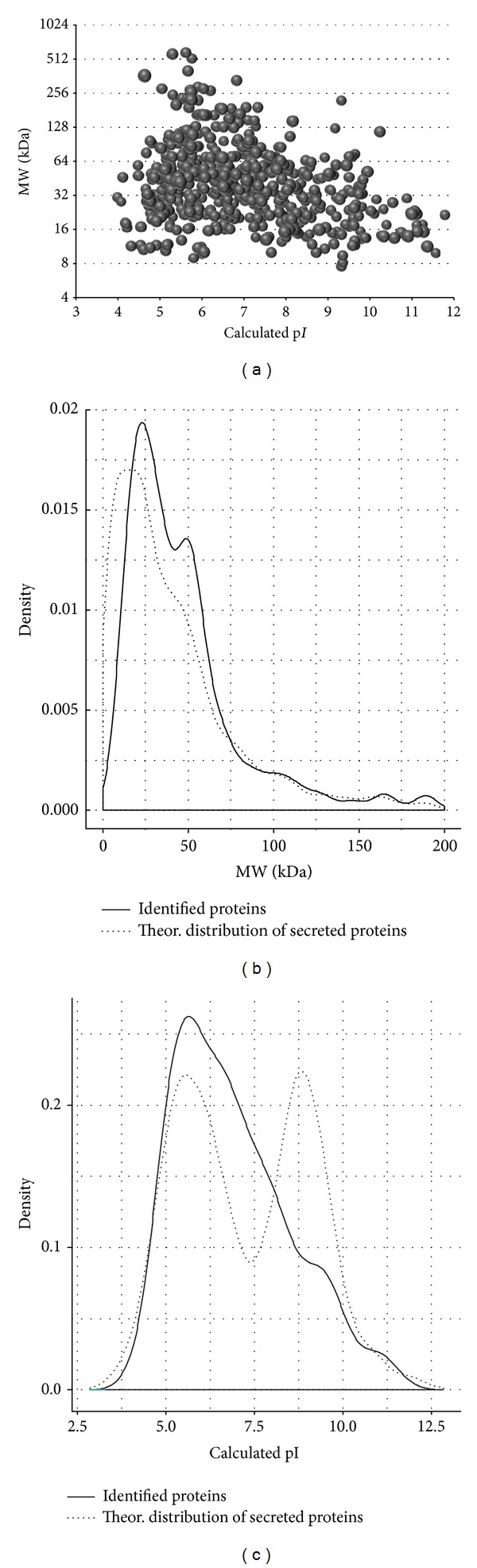
Analysis of the MW and pI distribution of the proteins being identified in WF of soft tissue and femoral bone defects. (a) Simulated 2D gel and distribution of the identified proteins compared to all proteins currently known to be secreted (according Gonzales et al. [24]) in respect to (b) the MW and (c) the pI.

The 600 unambiguously identified proteins which were detected in at least 2 biological replicates in either the soft tissue or the femoral bone defect were tested for a bias in respect to physical and functional parameters. As a first aspect it was checked whether the utilization of the 100 kDa cutoff dialysis membrane led to depletion of low molecular weight proteins or proteins being highly acid or basic. An artificial 2D gel was generated, in which all detected proteins were plotted according the calculated molecular weights and pI values (Figure 3(a)). According to these analyses proteins covering a MW range of less than 10 kDa to more than 250 kDa and a pI range from less than 4 to more than 11 were identified. Interestingly even very small proteins (<10 kDa) such as ATP synthase subunit E (8.2 kDa) and the immunoglobulin epsilon receptor subunit gamma (9.8 kDa) could be detected.

As a reference dataset all human proteins which are secreted according to an analysis of Gonzales et al. [24] were compared to the identified proteins in order to check for significant differences in the distributions (Figure 3(b)). As typical for GeLC-MS analysis a strong underrepresentation of low molecular weight proteins (<15 kDa) was observed. In contrast proteins with a higher molecular weight were detected with the same frequency as expected based on the reference dataset. In respect to the pI values the distribution of the detected proteins differed clearly from the distribution of the reference dataset (Figure 3(c)).

### 3.5. Coverage of Wound Process Related Proteins and Potential Biomarkers

All 600 proteins which were identified in WF of soft tissue or femoral bone defects were clustered according to their GO classifications: “cellular component” and “biological function” (Supplementary Tables 4A and 4B). With 103 proteins (of the 483 proteins assigned to cellular compartments in the Database for Annotation, Visualization and Integrated Discovery (DAVID)) the fraction of extracellular proteins (for a complete list including the clustered proteins see Supplementary Table 4A) is well represented and about 1.7-fold enriched compared to the distribution of all annotated proteins.

**Figure 4:**
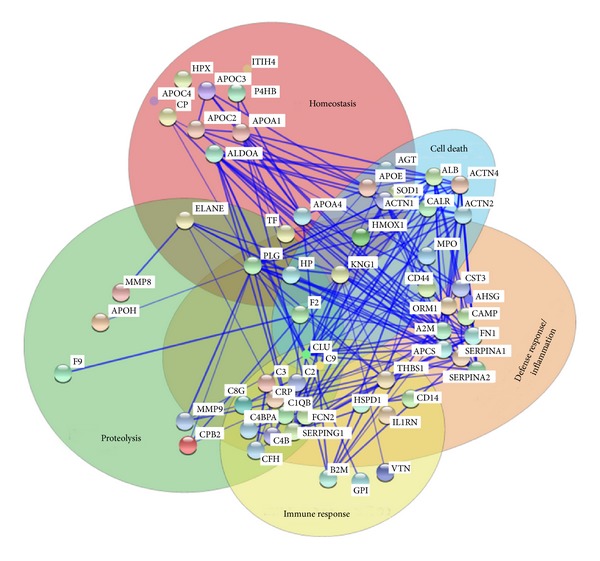
Protein-protein interaction network of secreted proteins being identified in soft tissue and femoral bone defects. The 60 proteins which were (i) identified in our study, (ii) annotated to be extracellularly located, and (iii) assigned to at least one molecular function were clustered according the annotated biological process. Connected proteins are experimentally determined protein-protein interaction partners.

In respect to the functional grouping the major protein clusters are “response to wounding” (63 of the 456 annotated proteins/3.5-fold enrichment), “homeostatic process” (58/2.3), “proteolysis” (57/1.6), “regulation of apoptosis” (57/2.1), “defense/inflammatory response” (55/2.7), vesicle-mediated transport (40/2.1), “acute inflammatory response” (25/7.6), and “response to oxidative stress” (24/4.8) (for complete list see Supplementary Table 4B). The same termini were also enriched if only detected secreted proteins were considered (Supplementary Table 4C). Interestingly, 60 of the 72 extracellular proteins which are assigned to at least one biological process could be clustered to at least one of the processes related to a wound healing phase (Figure 4). 18 proteins are assigned to homeostasis and blood coagulation (wound healing phase I), 35 proteins are assigned to inflammation or defense response (wound healing phase II), and 19 are related to proteolysis and remodeling (wound healing phase III and IV). Additionally, four proteins which are exclusively assigned to regulation of cell death were included.

In our analysis several proteins were covered which were previously discussed as direct biomarker candidates such as the matrix metalloproteases MMP-8 and MMP-9, the oxidative stress marker myeloperoxidase (MPO), several annexins and S100 proteins, 15 different serin proteases (e.g., pronase 3 or neutrophilic elastase 2) or serin protease inhibitors, and 9 complement factors (Table 1). Cytokines or chemokines were not detected in the proteomic analysis of the WF; however, cytokine related proteins (e.g., IL-1 antagonist) and more than 50 proteins related to inflammation could be covered (Supplementary Table 3B). Analogously, more than 20 proteins known to be induced as response to oxidative stress (e.g., superoxide dismutases, peroxiredoxins, and glutathione peroxidases) could be detected.

### 3.6. Differentially Abundant Proteins in WF of Femoral Bone versus Soft Tissue Defect

An ultimate goal of WF analysis is the detection of proteins being specifically enriched or exclusively present in samples derived from defined defect scenarios.

For this purpose all proteins were determined which are (i) either reproducibly detected in the femoral bone defect but were covered in less than two replicates of the soft tissue defect or (ii) determined to be at least 10 times more abundant in bone defect or soft tissue defect samples. Nine proteins fulfill the first or second criteria, respectively. Among those there are three proteases (carboxypeptidase B2, proteasome subunit alpha type-6, and proteasome subunit alpha type 7), one protease inhibitor (protein Z-dependent protease inhibitor), and two S100 proteins (S100-A8 and S100-A9) which might be candidates to monitor femoral bone defect specific processes (Supplementary Table 5).

## 4. Discussion

### 4.1. Discontinuous Sampling of Wound Fluid

Due to the low amounts and concentrations of proteins, the high dynamic range of the analyte concentrations in WF, and the difficulties in collecting the WF in a minimal-invasive way, proteomic studies are challenging. In previous proteomic studies mainly discontinuous sampling methods were used such as microsolid phase extraction [29] or exudates [30]. It has been demonstrated that using these methods combined with protein enrichment and purification the detection of more than 120 proteins is possible [30]. However, an ideal sampling technique of proteins from wound fluids would be minimal invasive, would allow continuous sampling, would yield a sufficient amount of protein without the need for protein purification or enrichment, and would allow comprehensive coverage of the WF proteome.

Here we present the first proteomic study of wound fluid using adsorption by microdialysis catheter as a sampling technique and evaluated the potential of this approach. This investigation revealed three major findings. (i) microdialysis catheters can not only be used to continuously sample metabolites, cytokines, and chemokines in the dialysate but also be used to simultaneously extract higher molecular weight proteins (>10 kDa) by adsorption in sufficient amounts and purity for a GeLC-MS/MS analysis. (ii) 600 proteins could be identified including a high percentage of proteins being involved in wound healing phases I, II, and III as well as several previously reported biomarkers for diagnosis of adverse effects in wound healing. (iii) Different defect types can be studied and abundance differences can be investigated.

### 4.2. Analysis of the Dialysate

Even if discontinuous sampling of larger proteins from WF is an option for* in situ* sampling, the continuous sampling is still preferable since this allows a time-dependent analysis. Using microdialysis catheters with ultralarge pore size (MW cutoff of 3 MDa) it has been demonstrated that it is possible to extract even high molecular weight proteins [18]. However, as reported by Gill et al. the estimated recovery of serum albumin was about 10% and for a successful proteome analysis desalting and enrichment is necessary—an additional depletion of high abundant serum proteins is a further option to improve the proteome coverage [15]. Nevertheless, even though the reproducibility of the quantification was excellent and the number of detectable proteins could be doubled after depletion of the 6 most abundant proteins, still only 80 proteins could be detected [15].

For the femoral defect which was investigated in this study the application of a catheter of an ultralarge pore size was technically not possible since this resulted in a high buffer influx into the wound area. Using a catheter with a MW cutoff 100 kDa it was possible to reproducibly quantify 15 proteins including serpin c1 and complement C3 which play dominant roles in blood coagulation and immune response, respectively. Furthermore it has been shown that microdialysis is capable of sampling cytokines in the dialysate using our experimental setup [13], proving that this method actually can provide molecular information from processes being relevant for wound healing. More specifically Förster et al. analyzed the cytokines IL-6, TGF-*β*1 by ELISA. Thereby it was also possible to obtain quantitative information of the first 24 h after surgery [13]. These analyzed cytokines are only a subset of those smaller proteins (<20 kDa) which are of potential interest. For example, the detection of many other cytokines such as IL-1, IL-8, and TNF-*α* is of high interest. For several cytokines ELISA assays are already established; however, many low molecular proteins still either lack a specific ELISA assay or are in their abundance too low so that they are not feasible for detection by either ELISA or global LC-MS/MS. The development of targeted mass spectrometry such as the selected reaction monitoring (SRM) or the parallel reaction monitoring (PRM) approach might soon be capable of detecting concentrations below the normal detection limit in global proteome analyses and thus will increase the number of detectable low molecular weight proteins in dialysates. In addition these approaches can also be used for absolute quantification [31].

Another so far neglected aspect of the dialysate is the metabolomic analysis. It is well accepted that, first, the metabolomic niche has a strong influence on cell differentiation and in particular on stem cells and immune cells [32] and, second, that many types of cells secrete specific metabolites depending on their physiological state [33, 34]. In two recent studies microdialysis in combination with metabolomics was used to assess the muscle interstitium fluid in the human trapezius myalgia [35] and for monitoring the effects of radiotherapy of glioblastomas. In the first case the dialysate was analyzed by GC-MS and multivariate analysis yielded significant differences from the myalgic muscle in comparison to the control. In the latter case both detection and quantification were performed by GC-MS and NMR and resulted in the detection of molecular patterns associated with therapy. In this study we were able to quantify more than 150 metabolites from the same samples which were also investigated by proteomics including amino acids, hexoses, acylcarnitines, and phospho- and sphingolipids. The determined sensitivity of the analysis, the possibility to perform this metabolite analysis in high throughput, and the experimental setup would allow sampling of metabolites every 10 min to study time-dependent metabolite abundances.

### 4.3. Combination of Continuous and Discontinuous Sampling

In wound healing 4 phases are typically distinguished which are (i) homeostasis, (ii) inflammation, (iii) proliferation, and (iv) remodeling. We have directly detected proteins from the processes of cellular homeostasis and blood coagulation (phase I), and acute inflammatory response and immune response (phase II). Furthermore proteins which are functionally related to the response to oxidative stress, cellular apoptosis, and especially proteolysis are well covered. As potential protein biomarkers which may allow the monitoring of discrepancies during the different phases mainly cytokines and proteases were discussed. Especially IL-1, IL-6, IL-8, and TNF-*α* were found to be increased in chronic wounds [36]. Additionally increased (metallo-)proteases levels (e.g., MMP-7 and MMP-2 can be used for prediction of wound healing outcome of traumatic war wounds [5]), protease/inhibitor ratios (e.g., MMP9/TIMP ratio [37]), and the expression level of myeloperoxidase as well as of procalcitonin have been reported. Furthermore collagen and S100A8 and A9 protein levels were previously shown to be more abundant in nonhealing wounds [38]. As nonprotein markers, the tissue bacterial level, the level of oxidative species, and the level of volatile organic compounds were shown to have predictive power as reviewed by Yussof et al. [36].

In an initial study by Förster et al. the potential to continuously sample cytokines by microdialysis and quantify them in the microdialysis dialysate has already been shown and can be extended to quantify further cytokines [13].

In cases such as bone wound fluid analysis where the protein recovery from the dialysate is not satisfactory in terms of quality or quantity to perform a comprehensive proteome analysis, the study being presented here gives an example of how to use the adsorption to the microdialysis catheter surface for an additional analysis. Using this complementary approach allows simultaneously the continuous sampling of cytokines, metabolites, and other small proteins in the dialysate in combination with discontinuous sampling of larger proteins by adsorption. The achieved coverage of more than 600 identified proteins is, to the best of our knowledge, much higher than that in all previous proteomic studies of bone defect wound fluids. Therefore, it provides a blueprint for secreted proteins in wound fluid. The observed underrepresentation of low molecular weight proteins might be due to a combination of two effects: (i) the dialysis is currently optimized to maximize the recovery of small proteins in the dialysate and not in the adsorbate and (ii) the applied GeLC-MS/MS approach was not especially optimized to detect low molecular weight proteins. The first reason could be minimized by using lower molecular weight cutoffs or by a reduction of the BSA contentment of the dialysate. The second reason can be addressed by switching from a GeLC-MS/MS to an 2D-LC-MS/MS analysis or by using gels which are optimized to separated low molecular weight proteins as recently demonstrated [39].

The additional gain is further emphasized by the fact that many proteins which are reported to have predictive or diagnostic potential for wound healing are among the detected proteins (see Table 1). Furthermore the most relevant pathways in wound healing could be covered (see Figure 4).

## 5. Conclusion

In this study, we could show (i) that adsorption based sampling of proteins with microdialysis catheters allows to collect, enrich, and purify proteins in a sufficient amount for a proteome analysis, (ii) that more than 600 proteins can be detected in different defects, (iii) that more than 100 secreted proteins can be extracted including many proteins which have previously been demonstrated to have diagnostic or predictive power for the wound healing state, (iv) that there is only a low bias regarding the MW (loss of small proteins) and pI (underrepresentation of basic proteins), and (v) that the continuous sampling of metabolites and cytokines and discontinuous sampling of proteins can be combined efficiently and provide complementary information.

In conclusion in one experimental setup abundance profiles of metabolites and of selected low molecular weight proteins (dialysate analysis) and a comprehensive proteome profile after accumulation during a defined sampling time (analysis of the adsorbate) can be obtained from the same defects.

## Supplementary Material

Table S1: Quantification results of the 163 selected metabolites which were measured in the dialysates of the femoral bone and soft tissue defect.
Table S2: Qualitative, quantitative, and functional information about all proteins being quantified in the dialysates in at least 2 replicates of the femoral bone or soft tissue defect
Table S3: Qualitative, quantitative, and functional information about all proteins being quantified in the adsobates in at least 2 replicates of the femoral bone or soft tissue defect.
Table S4: List of the main gene ontology clusters of (A and C) biological processes, (B) cellular compartments covered by the all identified proteins (A) and all secreted proteins (C).
Table S5: List of the proteins being either reproducible detected in the femoral bone defect but were only covered in maximum one replicate of the soft tissue defect or (ii) which were detected with a more than 10 times altered intensity in bone compared to soft tissue samples.

## Figures and Tables

**Table 1:** Potential biomarkers being detected in WF samples of the soft tissue or femoral bone defect.

Description	UniProt accession	Gene ID	Description	MW [kDa]	Calc. pI	Max. Mascot score	Number of unique peptides	% sequence coverage	References	Further related proteins
Complement Factors	Q6P6G4	C1qb	Complement C1q subcomponent subunit B	26.6	8.8	42.1	2	11	Cazander et al., 2012 [40]	C1qb, C2, C3, C4, C4b, C8g, and C9
	G3V615	C3	Complement C3	186.2	6.5	2128.3	87	57	Cazander et al., 2012 [40]	

Serin proteases and inhibitors	Q5RKH1 Q3T1J1	Prtn3 ELANE	Protein Prtn3 elastase 2, neutrophil	27.7 29.5	8.0 9.3	48.9 96.1	2 7	16 28	Eming et al., 2007 [41] Buchstein et al., 2009 [42]	A1m, A2m, SERPINA4, SERPINC1, SERPINF2, Ambp, C3, C4, C4b, C8g, Itih1, Itih3, and Itih4

Cytokine receptor antagonists	P20761	Il1rn	Interleukin-1 receptor antagonist protein	20.3	6.9	51.5	3	17	Ishida et al., 2006 [43]	

Matrix metallopeptidases	P08011	Mmp8	Matrix metallopeptidase 8	53.2	6.6	42.7	5	16	Gutiérrez-Fernández et al., 2007 [44]	Anpep, Cpb2, Hpx, and Lta4h
	G3V7D0	Mmp9	Matrix metallopeptidase 9	78.5	6.3	65.6	4	8	Reiss et al., 2010 [45]	

Oxidoreductases	D3ZYK8	Mpo	Myeloperoxidase (mapped)	51.9	9.9	798.4	9	66	Moor et al., 2009 [7]	Gpx1, Gpx3, Gsr, HMOX1, Impdh2, Lbr, Ldha, Ldhb, Ldhc, Mdh1, Mdh2, Mpo, Mtco2, Ndufv2, Nos2, Pgd, Prdx1, Prdx2, Prdx6, Sod1, Sod2, Sod3, Tkt, and Txn

S100 proteins and annexins	Q6B345	S100a8	Protein S100-A8	10.2	6.1	426.6	7	87	Thorey et al., 2001 [38]	S100a11, Anxa2, Anxa3, Anxa4, Anxa5, Anxa6, Anxa7, and Anxa11
	P50115	S100a9	Protein S100-A9	13.1	7.5	375.5	15	84	Wyffels et al., 2010 [20]	
	G3V7W7	Anxa1	Annexin A1	38.8	7.3	1750.0	33	75	Leoni et al., 2013 [46]	

Proteins which were reported as biomarker candidates and could be identified in at least 2 samples of the soft tissue or femoral bone defect are grouped according to their annotated protein classes. Additionally, further proteins being identified and belonging to the same protein classes are listed.

## Abbreviations

WF: Wound fluid
GeLC-MS/MS: In-gel separation and proteolytic digestion followed by liquid chromatography-tandem mass spectrometry.
